# Combinations of Oseltamivir and T-705 Extend the Treatment Window for Highly Pathogenic Influenza A(H5N1) Virus Infection in Mice

**DOI:** 10.1038/srep26742

**Published:** 2016-05-25

**Authors:** Bindumadhav M. Marathe, Sook-San Wong, Peter Vogel, Fernando Garcia-Alcalde, Robert G. Webster, Richard J. Webby, Isabel Najera, Elena A. Govorkova

**Affiliations:** 1 Department of Infectious Diseases, St. Jude Children’s Research Hospital, Memphis, Tennessee, United States; 2 Roche Pharma Research and Early Development, Infectious Diseases, Roche Innovation Center Basel, F. Hoffmann-La Roche Ltd., Basel, Switzerland

## Abstract

Current anti-influenza therapy depends on administering drugs soon after infection, which is often impractical. We assessed whether combinations of oseltamivir (a neuraminidase inhibitor) and T-705 (a nonspecific inhibitor of viral polymerases) could extend the window for treating lethal infection with highly pathogenic A(H5N1) influenza virus in mice. Combination therapy protected 100% of mice, even when delayed until 96 h postinoculation. Compared to animals receiving monotherapy, mice receiving combination therapy had reduced viral loads and restricted viral spread in lung tissues, limited lung damage, and decreased inflammatory cytokine production. Next-generation sequencing showed that virus populations in T-705–treated mice had greater genetic variability, with more frequent transversion events, than did populations in control and oseltamivir-treated mice, but no substitutions associated with resistance to oseltamivir or T-705 were detected. Thus, combination therapy extended the treatment window for A(H5N1) influenza infection in mice and should be considered for evaluation in a clinical setting.

Highly pathogenic avian influenza (HPAI) A(H5N1) viruses remain a serious threat to wild and domestic poultry and have pandemic potential for humans because of the low level of pre-existing immunity in the population. These viruses have undergone substantial evolution and have expanded geographically since emerging in Asia, with clades 2.3.2.1 and 2.3.4 becoming the predominant lineages^1^,^2^. Since 2003, HPAI A(H5N1) viruses have caused sporadic disease in humans, and 846 laboratory-confirmed human cases were reported from 2003 through May 2015^1^. Human infections are characterized by fatality rates of approximately 60% and severe manifestations^2^,^3^. Currently, there is no evidence of sustained, human-to-human spread of HPAI A(H5N1) viruses, though their cocirculation with seasonal influenza viruses among humans and animals could lead to coinfections, reassortment, and the emergence of novel viruses with pandemic potential^4^,^5^. Importantly, the threat from HPAI viruses is not limited to the A(H5N1) subtype; the A(H5N2), A(H5N6), and A(H5N8) subtypes were recently detected in North America and Asia^6^,^7^.

Vaccination and antiviral therapy represent the key options for controlling influenza virus infections in humans. Although candidate A(H5N1) vaccines for different HA clades have been developed and approved for national stockpiling, they are characterized by poor immunogenicity, though this can be enhanced by a prime-boosting strategy and oil-in-water adjuvants^8^,^9^. At present, only a single class of drugs is approved by the United States FDA and is clinically available for treating influenza, i.e., neuraminidase (NA) inhibitors (NAIs) (oseltamivir, zanamivir, and peramivir). NAIs are effective against all subtypes of influenza viruses and remain the primary treatment option. Although adamantanes (amantadine, rimantadine), that target M2 ion channel, are FDA-approved against infection caused by influenza A viruses, they are not recommended by the United States CDC for the prophylaxis and treatment of influenza due to high frequency of drug-resistance among circulating influenza A viruses. Moreover, influenza B viruses are not susceptible to adamantanes^10^. Treatment with NAIs is effective when dosing is initiated within 48 h after the onset of symptoms; however, the potential emergence and circulation of NAI-resistant variants could further limit the treatment options. Combination therapy with 2 or more multi-target antiviral drugs could potentially improve the outcome of influenza infections; decrease the emergence of drug-resistant variants^11^; and limit viral spread and, subsequently, cytokine release and immunopathogenic changes. Combination therapy has proved a successful strategy for controlling other viral infections, such as infections with human immunodeficiency virus (HIV-1) and hepatitis B and C viruses^12^,^13^,^14^. For influenza, combinations of NAIs with adamantanes (amantadine, rimantadine), ribavirin, or immunomodulatory drugs have demonstrated additive or synergistic drug interactions in cell culture and increased survival rates in a mouse model^15^,^16^,^17^,^18^. However, limited information is available on the treatment efficacy of combination therapy in the clinical setting. In a randomized, controlled trial of hospitalized patients, a combination of nebulized zanamivir and oral rimantadine had slightly greater antiviral efficacy than did monotherapy, and the emergence of rimantadine resistance was prevented^19^. A triple combination of oseltamivir, amantadine, and ribavirin is currently undergoing randomized, controlled clinical trials in high-risk patients^20^.

Several novel antiviral drugs are currently in development for controlling influenza and may offer new options for combination therapy. Of particular interest is the nucleoside analog T-705 (favipiravir), a nonspecific inhibitor of the RNA-dependent RNA polymerase of influenza virus that is active against a broad range of influenza A, B, and C viruses, including HPAI A(H5N1) and newly emerging A(H7N9) viruses^21^,^22^. Delayed treatment (24 h after virus exposure) with oseltamivir and T-705 combinations increased survival of mice infected with A/duck/Minnesota/1525/1981 (H5N1) influenza virus as compared to both monotherapies^23^. T-705 was approved in Japan in 2014 for stockpiling for pandemic preparedness only, not yet for the treatment of seasonal influenza, and is undergoing phase III clinical trials in the United States^24^,^25^.

The main obstacle to successful NAI therapy of influenza is the requirement for early initiation of drug administration, ideally within 48 h after the onset of symptoms. Most patients do not present to the clinic within this timeframe, as has been shown for both A(H5N1) infections and severe seasonal influenza illness^26^,^27^,^28^,^29^,^30^. Thus, treatment regimens that can extend the window of efficacy are urgently needed and would significantly expand the options for clinical management of severe influenza. Accordingly, we studied whether combination therapy with oseltamivir and T-705 extended the window of treatment efficacy against lethal infection of mice with HPAI A(H5N1) virus.

## Results

### Morbidity and survival in mice lethally challenged with influenza A(H5N1) virus

**Figure 1:**
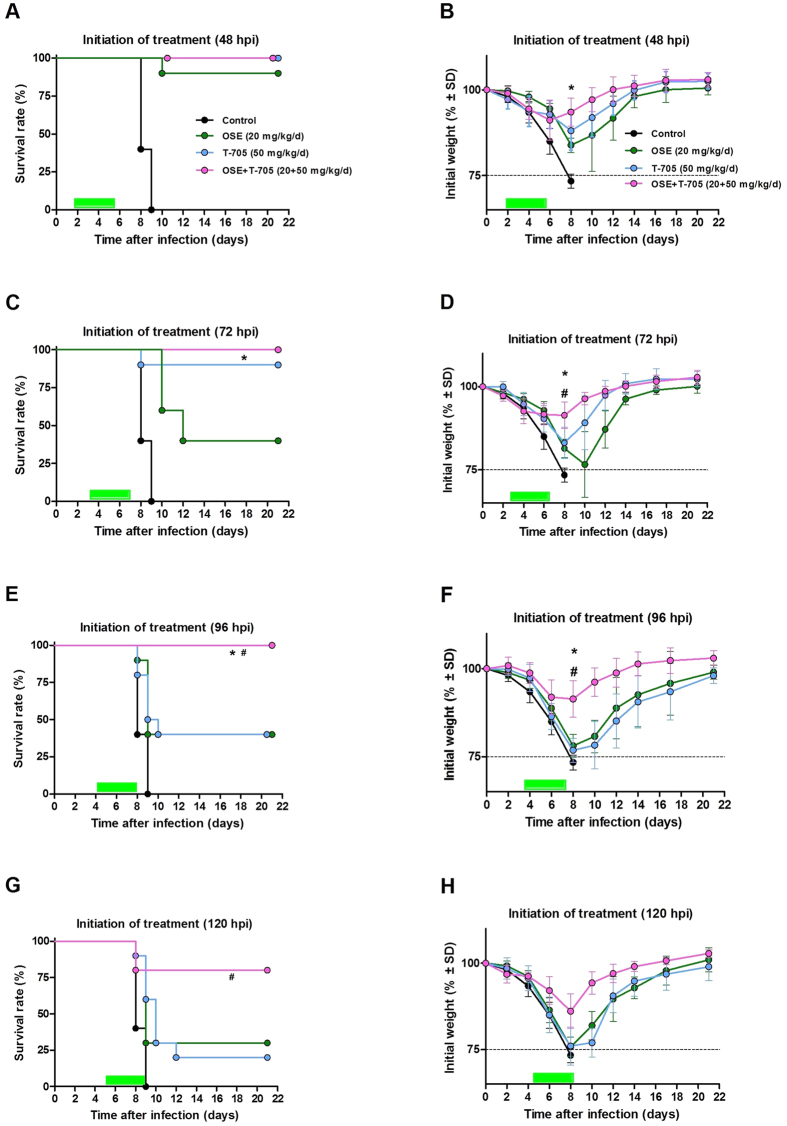
Survival and weight change in A(H5N1) virus–inoculated mice treated with oseltamivir, T-705, or their combination. Female 6- to 8-week-old BALB/c mice (*n* = 10/group) were lightly anesthetized with isoflurane and inoculated intranasally with 10 MLD_50_ of A/Turkey/15/2006 (H5N1) influenza virus. Mice were treated with oseltamivir (20 mg/kg/day), T-705 (50 mg/kg/day), or their combination starting at 48, 72, 96, or 120 hpi. The drugs were administered twice daily for 5 days by oral gavage. The green bars indicate the period of drug administration, and dotted line indicates endpoint for mortality (75% of initial weight). Control animals were treated with vehicle (water and ORA-PLUS, 1:1) on the same schedule. The graphs show the survival (**A,C,E,G**) and weight loss (**B,D,F,H**) of mice when treatment was initiated 48 (**A,B**), 72 (**C,D**), 96 (**E,F**), and 120 (**G,H**) hpi. *P < 0.05, compared between combination and oseltamivir monotherapy groups; ^#^*P* < 0.05, compared between combination and T-705 monotherapy groups. Probabilities for survival were determined by log rank test and for difference in weights by 1-way ANOVA. Abbreviations: OSE, oseltamivir; OSE + T-705, oseltamivir and T-705 combination.

The survival of animals undergoing antiviral treatment is the major determinant of drug efficacy. Control mice exhibited progressive weight loss (Fig. 1B,D,F,H), an essential marker of morbidity in influenza virus infection, and all succumbed to infection between 8 and 9 dpi. Weight loss was less pronounced when T-705 treatment was initiated at 48 or 72 hpi. Initiating combination therapy with oseltamivir and T-705 up to 96 hpi resulted in less weight loss than with either drug monotherapy. At 8 dpi, significant differences (*P* < 0.05) were observed between the weight loss in animals receiving combination therapy and that in mice receiving monotherapy.

Treatment with oseltamivir protected 90, 40, 40, and 30% of animals when initiated at 48, 72, 96, and 120 hpi, respectively (Fig. 1A,C,E,G). Treatment with T-705 provided complete protection (100% survival) against A(H5N1) virus when administered 48 hpi (Fig. 1A), but the level of protection decreased when treatment began at 72, 96, or 120 hpi, which resulted in 90, 40 and 20% survival, respectively (Fig. 1C,E,G). In contrast, combination therapy provided complete protection (100% survival) when initiated at 48, 72, or 96 hpi (Fig. 1A,C,E), and 80% of mice survived when initiation was delayed until 120 hpi (Fig. 1G). Statistically significant increases (*P* < 0.01) in survival were seen on comparison of the groups treated with the oseltamivir, T-705 and their combinations to the control group at all treatment initiation times. The combination treatment significantly increased (*P* < 0.05) the survival of mice as compared to that of mice that received oseltamivir when treatment was initiated at 72 and 96 hpi, and to that of mice that received T-705 when treatment was initiated at 96 and 120 hpi. Thus, combination therapy conferred greater survival benefits than did monotherapy and extended the window of treatment efficacy. The level of protection depended on the time of treatment initiation, with a more pronounced effect being observed when treatment began closer to the time of virus inoculation.

### Influenza A(H5N1) virus replication in lungs of mice

**Figure 2:**
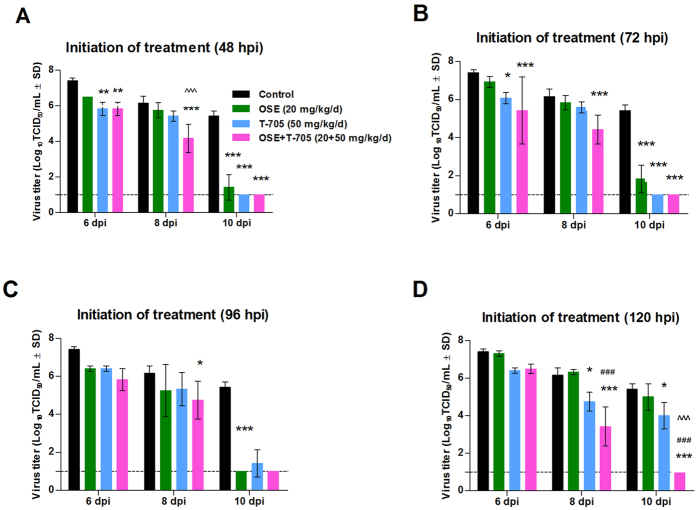
Lung virus titers in A(H5N1) virus–inoculated mice treated with oseltamivir (20 mg/kg/day), T-705 (50 mg/kg/day), or their combination. BALB/c mice were treated as described in the legend for Fig. 1. Virus titers were determined in the lungs of mice (*n* = 3/group) at 6, 8, and 10 dpi by 50% TCID_50_ assays on MDCK cells. The bars represent the mean virus titers in mouse lungs when treatment was initiated 48 (**A**), 72 (**B**), 96 (**C**), or 120 (**D**) hpi. *P < 0.05; **P < 0.01; and ***P < 0.001, compared between combination and control groups; ^###^*P* < 0.001, compared between combination and oseltamivir monotherapy groups; and ^^^^^*P* < 0.001, compared between combination and T-705 monotherapy groups. Probabilities were determined by 2-way ANOVA. Abbreviations: OSE, oseltamivir; OSE + T-705, oseltamivir and T-705 combination.

Oseltamivir treatment did not cause a significant reduction in virus lung titers at 6 or 8 dpi, irrespective of the time of treatment initiation. Only at 10 dpi, virus lung titers in oseltamivir-treated mice were significantly lower (*P* < 0.001) than those in controls, and this effect was observed in animals that received oseltamivir starting at 48, 72, or 96 hpi (Fig. 2A–C). In contrast, treatment with T-705 significantly reduced virus titers at 6 dpi compared to those in controls when treatment was initiated at 48 hpi (*P* < 0.01) and 72 hpi (*P* < 0.05) (Fig. 2A,B). In the combination therapy groups, virus titers were significantly lower than those in control animals on each day tested with statistical significance ranging between *P* < 0.05, and *P* < 0.001 when treatment was initiated at 48, 72, 96 and 120 hpi (Fig. 2). Notably, combination therapy completely eliminated virus replication in the lungs at 10 dpi, even when treatment was initiated as late as 120 hpi. Taken together, these results show that combination treatment with oseltamivir and T-705 was more effective than either of the monotherapies at reducing the virus load at the site of infection.

### Histologic changes in mouse lung tissues

**Figure 3:**
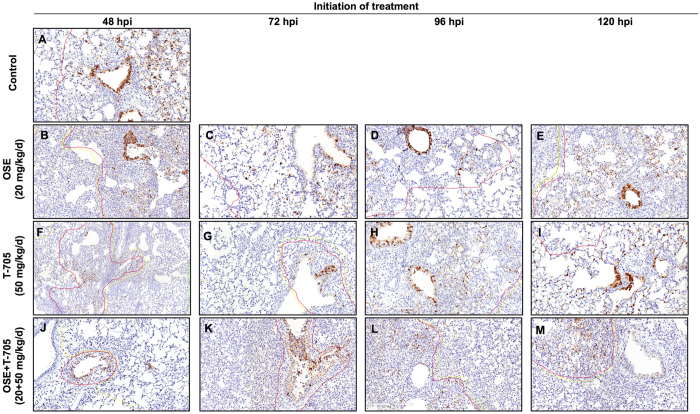
Histologic changes in the lungs of A(H5N1) virus–inoculated mice treated with oseltamivir (20 mg/kg/day), T-705 (50 mg/kg/day), or their combination. BALB/c mice were treated as described in the legend for Fig. 1. Pulmonary lesions were evaluated at 8 dpi (*n* = 2/group). The red lines designate areas with antigen-positive cells (active infection); the yellow lines outline areas with lesions but negligible antigen (inactive infection). In control mice (**A**), the lesions involved almost entire lobes and were characterized by widespread infection of bronchiolar epithelium, type II pneumocytes, and alveolar macrophages. In mice receiving oseltamivir monotherapy (**B–E**), active virus infection was generally restricted to airway epithelium and adjacent parenchyma (**B**), but when treatment was delayed until 72, 96, or 120 hpi (**C–E**), a broad band of infected type II pneumocytes surrounded the central lesions, which consisted of thickened septa and antigen-positive bronchioles. In mice receiving T-705 monotherapy (**F–I**), active virus infection was generally restricted to a few terminal airways and a narrow leading edge surrounding some lesions, but most antigen-positive cells in pulmonary lesions were only lightly stained. In mice receiving combination therapy (**J–M**), there was a marked reduction in both the extent of lesion development and the intensity of antigen staining. The lesions in combination therapy lungs were generally restricted to the peribronchiolar parenchyma, and antigen-positive cells were relatively uncommon and only lightly stained. Magnification, ×20. Abbreviations: OSE, oseltamivir; OSE + T-705, oseltamivir and T-705 combination.

The spread of A(H5N1) virus infection was most extensive in the lungs of control animals (Fig. 3A), which were characterized by extensive inflammation and necrosis of the bronchiolar epithelium, perivascular and peribronchiolar inflammation and edema, and the destruction and loss of type II pneumocytes in the alveoli. In the control lungs, a broad zone of strongly positive type II pneumocytes along the leading edge of lesions represented recently and actively infected alveoli. In animals that received oseltamivir, the extent and severity of the lesions and active infection were moderately reduced when treatment was initiated at 48 hpi (Fig. 3B). Viral antigen–positive type II pneumocytes were abundant at the periphery of lesions when treatment was initiated at 48 or 72 hpi but were spread throughout lesions when treatment was delayed until 96 or 120 hpi. In mice that received T-705 at 48 or 72 hpi (Fig. 3F,G), the pulmonary lesions were mild to moderate, with virus–positive cells scattered throughout the affected areas. The zone of strongly positive type II pneumocytes along the leading edge of lesions was narrow in lungs when T-705 treatment was initiated at 48 or 72 hpi, but it was characterized by broad leading edges containing abundant antigen-positive cells when treatment was delayed until 96 or 120 hpi. In contrast, combination therapy with oseltamivir and T-705 markedly reduced the extent and severity of pulmonary damage resulting from A(H5N1) virus infection when treatment was initiated at 48 hpi and caused only mild to moderate lesions when treatment was initiated at 72, 96, or 120 hpi (Fig. 3K–M). The lungs of mice receiving combination therapy did not have a zone of strongly positive type II pneumocytes adjacent to the leading edge of lesions, indicating the absence of recently infected cells.

**Figure 4:**
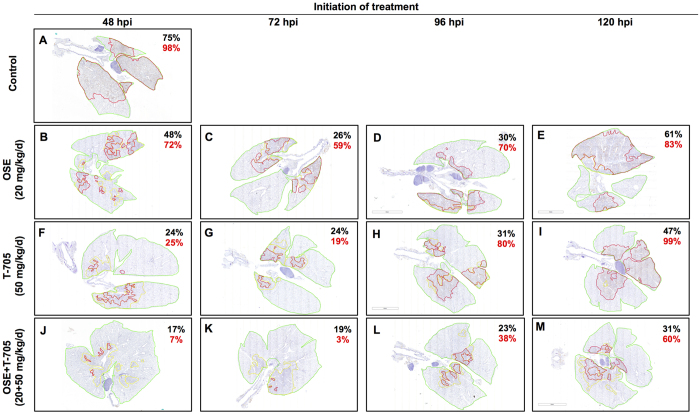
Histomorphometry of the lung tissues of A(H5N1) virus–inoculated mice treated with oseltamivir (20 mg/kg/day), T-705 (50 mg/kg/day), or their combination. BALB/c mice were treated as described in the legend for Fig. 1. For each mouse evaluated, one representative slide that included all the lung lobes was used. The red lines designate areas with antigen-positive cells (active infection); the yellow lines outline areas with lesions but negligible antigen (inactive infection). The total lesion area (active and inactive infection) is indicated as the percentage of the total lung field examined and is shown in black, and active infection is indicated as the percentage of the total lesion area and is shown in red. In control mice (**A**), active infection (indicated as a percentage) was extensive throughout the lungs. In mice treated with either oseltamivir (**B–E**) or T-705 (**F–I**) monotherapy, the extent of pulmonary lesions was less than in controls. When initiated at 48 or 72 hpi, T-705 therapy (**F,G**) was more effective than oseltamivir therapy (**B,C**). Combination therapy markedly reduced the extent of pulmonary lesions at all assessed time points (**J–M**). Magnification, ×2. Abbreviations: OSE, oseltamivir; OSE + T-705, oseltamivir and T-705 combination.

Histomorphometry was employed to better characterize the progression of A(H5N1) virus infection in the lungs and to evaluate the efficacy of therapies at restricting virus spread when initiated at selected time points. As expected, control mice infected with A(H5N1) virus had the highest percentage (98%) of lung lesions showing active infection (Fig. 4A). Compared to controls, oseltamivir reduced the extent of both the total lesions and the actively infected areas in all treatment groups (Fig. 4B–D). However, T-705 was even more effective than oseltamivir at reducing the extent of pulmonary involvement and virus spread when treatment was initiated at 48 or 72 hpi. The treatment efficacy of T-705 was less pronounced when treatment was delayed until 96 or 120 hpi. Most importantly, combination therapy with oseltamivir and T-705 achieved an additional reduction in the extent of pulmonary lesions and the spread of virus infection than did monotherapy. The enhanced efficacy of combination therapy was clearly evident even when treatment was delayed until 96 or 120 hpi (Fig. 4L,M). Taken together, these findings indicate that combination therapy has beneficial effects in preventing virus spread and disease progression.

### Production of pulmonary cytokines and chemokines

**Figure 5:**
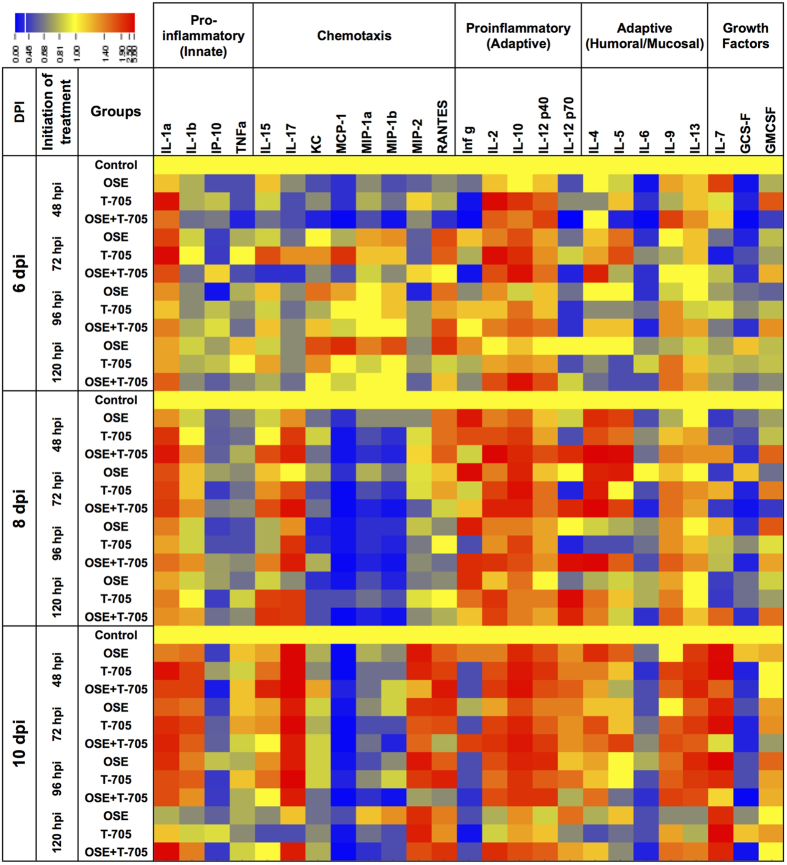
Pattern of cytokine and chemokine production in the lungs of A(H5N1) virus–inoculated mice treated with oseltamivir (20 mg/kg/day), T-705 (50 mg/kg/day), or their combination. BALB/c mice were treated as described in the legend for Fig. 1. Twenty-five cytokines and chemokines were assayed in lung homogenates (*n* = 3/group) at 6, 8, and 10 dpi by using a MYCTOMAG-70K-PMX MILLIPLEX^®^ MAP mouse cytokine/chemokine panel (Millipore). The concentration of each cytokine/chemokine tested was normalized to that for control A(H5N1) virus-infected animals (shown in yellow). The fold changes in the concentration of each cytokine or chemokine ranged from 0.00 (shown in blue) to 3.00 (shown in red). Abbreviations: OSE, oseltamivir; OSE + T-705, oseltamivir and T-705 combination.

**Figure 6:**
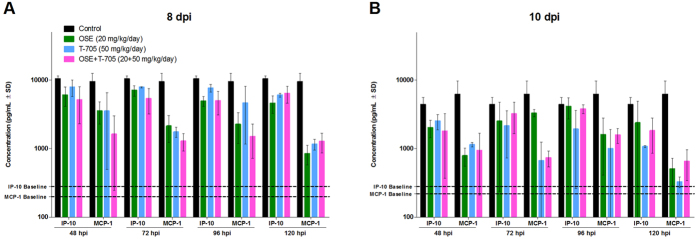
Levels of IP-10 and MCP-1 in the lungs of A(H5N1) virus–inoculated mice treated with oseltamivir (20 mg/kg/day), T-705 (50 mg/kg/day), or their combination. BALB/c mice were treated as described in the legend for Fig. 1. The concentrations of IP-10 and MCP-1 in lung homogenates (*n* = 3/group) at 6, 8, and 10 dpi were determined using a MYCTOMAG-70K-PMX MILLIPLEX^®^ MAP mouse cytokine/chemokine panel (Millipore). The bars indicate the mean concentration of IP-10 and MCP-1 (pg/mL) ± SD. The dashed lines indicate the chemokine concentrations in mock-infected mice. All treatment regimens significantly reduced (*P* < 0.01) the production of IP-10 and MCP-1, as compared to that in control mice, at 8 and 10 dpi when tested by 2-way ANOVA. Abbreviations: IP-10, inducible protein; MCP-1, monocyte chemotactic protein 1.

In humans and in animal models, immune-mediated pathology plays an important role in the severity of disease caused by HPAI A(H5N1) viruses. Therefore, we assessed the effect of treatment with oseltamivir, T-705, and their combination, when initiated at different time points, on the induction of 25 pro-inflammatory cytokines and chemokines in the lungs of mice infected with A(H5N1) virus (Fig. 5). Initiating monotherapy with oseltamivir or T-705 or combination therapy at 48 hpi decreased the induction of the pro-inflammatory cytokines at 6 dpi, with combination therapy having a more pronounced effect than either monotherapy. Similarly, combination therapy administered at 72 hpi decreased the induction of IFN-γ, IP-10, MCP-1, and TNF-α by 1 or 2 fold, as compared to their induction under either monotherapy. When combination therapy was initiated at 96 or 120 hpi, the induction of IFN-γ, IL-6, IP-10, and TNF-α was decreased by 1 fold, as compared to their induction under monotherapy. At 8 and 10 dpi, all treatment regimens reduced the induction of pro-inflammatory cytokines/chemokines (IL-6, IP-10, MCP-1, and MIP-1α), regardless of when treatment was initiated, with the combination therapy having a more pronounced effect than the monotherapies. The drug treatments had only moderate to no effect on TNF-α induction. With all treatment regimens, we detected a statistically significant reduction (*P* < 0.01) in the production of 2 chemokines (IP-10 and MCP-1), as compared to that in control mice, at 8 and 10 dpi (Fig. 6).

### Emergence of drug-resistant variants

To evaluate whether the drug treatment resulted in the emergence of oseltamivir- or T-705–resistant variants, we used next-generation sequencing (NGS) to examine the viral RNA extracted from mouse lung homogenates at 8 dpi, the latest time point at which virus was detectable across all treatment groups. Gene-specific primers were used to amplify PB1, PA and NA. Overall, PB1 and PA had a median coverage of 10,000 reads, whereas NA had a median coverage of 5,000 reads. However, some PB1 and PA samples in the combination therapy group amplified less efficiently than those in the monotherapy groups (Supplementary Fig. 1).

No dominant NA mutations associated with the oseltamivir-resistant genotype were detected in the virus populations in any experimental group. A low frequency (6.5%) of V116A substitution (N2 numbering) was identified in a single mouse in a group that received oseltamivir (initiated at 72 hpi). This substitution reportedly reduces the susceptibility of influenza A(H5N1) virus to oseltamivir^31^. Additionally, a single dominant (73.8%) T71I substitution in the stalk region of NA emerged in 1 of 3 mice in the combination group that received treatment at 48 hpi. No other treatment group–specific NA mutations were observed.

No dominant PA variants were detected in any animal, whereas dominant PB1 variants were detected in 3 mice that received combination therapy: G540E (99.57%), R584C (96.76%), and A659V (55.86%) (Supplementary Table 1). Because no T-705–resistant influenza viruses have yet been reported and no molecular markers for a T-705–resistant genotype are known, we compared the EC_50_ of the A/Turkey/15/2006 (H5N1) influenza virus used to inoculate the mice with that of the H5N1 virus–containing samples to determine if any of these mutations were associated with resistance. Importantly, the susceptibilities of viruses from mice under T-705 treatment were comparable to that of the inoculation virus, and the difference in EC_50_ did not exceed 2 fold (mean EC_50_, 14.9 ± 5.5 μM; data not shown). This suggests that the treatment did not lead to the emergence of T-705–resistant variants.

### Mutational landscape of the virus populations

Because T-705 targets the polymerase protein complex^25^,^32^, we hypothesized that the mutational profile of the virus population in animals treated with T-705 would differ from that of the virus population in the oseltamivir-treated and control mice. We looked for differences in the variability of these virus populations by using a single-reaction whole-genome amplification strategy^33^,^34^,^35^. We then used the Shannon Entropy Index (S_NT_) as a measure of virus genetic diversity. The S_NT_ was originally used in ecology to describe species richness and diversity but has been adapted to describe the diversity within a virus population based on sequence information^36^,^37^,^38^. In the present context, a higher S_NT_ reflects a more diverse sequence dataset (and, by extension, a highly mutated virus population) and vice versa.

The single-reaction whole-genome amplification approach resulted in uneven coverage across the 8 gene segments (data not shown). Using this amplification approach, the shorter genes (NP, NA, MP, and NS), which ranged in size from 863 to 1497 bp, were sequenced completely, with an average depth of 3867 to 6290 reads per position; however, complete sequence coverage was not achieved for the longer genes (HA, PA, PB1, and PB2), which ranged from 1701 to 2230 bp in length.

**Figure 7:**
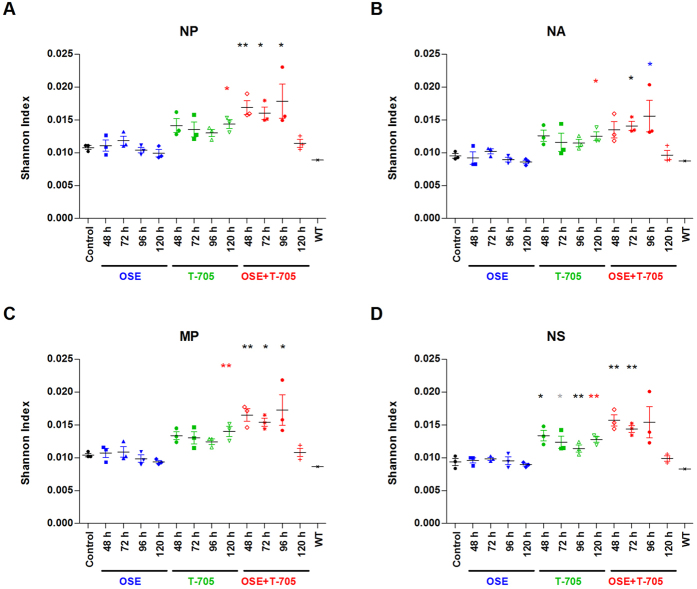
Virus population diversity in the lungs of A(H5N1) virus–inoculated mice treated with oseltamivir (20 mg/kg/day), T-705 (50 mg/kg/day), or their combination. BALB/c mice were treated as described in the legend for Fig. 1. The virus population diversity in the lung homogenates (*n* = 3/group) at 8 dpi was represented by the Shannon Entropy Index (S_NT_) obtained from the nucleotide deep-sequencing data for the following influenza genes: NP (**A**), NA (**B**), MP (**C**), and NS (**D**). *P < 0.05; **P < 0.01, and ***P < 0.001 when tested by 1-way ANOVA. Black * represents statistical significance when compared to control and oseltamivir, red * represents statistical significance when compared to control, oseltamivir, and combination group, blue * represents statistical significance when compared to oseltamivir, and gray * is significant only against control animals. Abbreviations: OSE, oseltamivir; OSE + T-705, oseltamivir and T-705 combination; NP, nucleoprotein; NA, neuraminidase; MP, matrix; WT, wild-type virus used for inoculation of mice.

There were no significant differences in S_NT_ among the 4 well-sequenced genes (NP, NA, MP, and NS) within the treatment group (Fig. 7), suggesting that the diversity is equally distributed across the 4 genes. The S_NT_s were significantly higher in the T-705 and combination therapy groups than in the control and oseltamivir-treated groups (*P* < 0.05), except when treatment was initiated at 120 hpi. At that time point, the S_NT_ for the combination therapy group was comparable to those for the control and oseltamivir-treated groups. This probably reflects the much-reduced viral titer in the samples from the combination treatment group, compared to that in the other samples (Fig. 2D). Although the combination therapy group tended to have a higher S_NT_ than did the T-705–treated group, this difference was not statistically significant. Additionally, there was a trend toward increased S_NT_ with earlier treatment initiation, but these differences were also not statistically significant. Collectively, these data suggest that T-705 treatment, whether administered alone or in combination with oseltamivir, results in a highly variable virus population, whereas treatment with oseltamivir alone does not; this is consistent with the known mechanism of action of the 2 drugs^39^,^40^,^41^.

**Figure 8:**
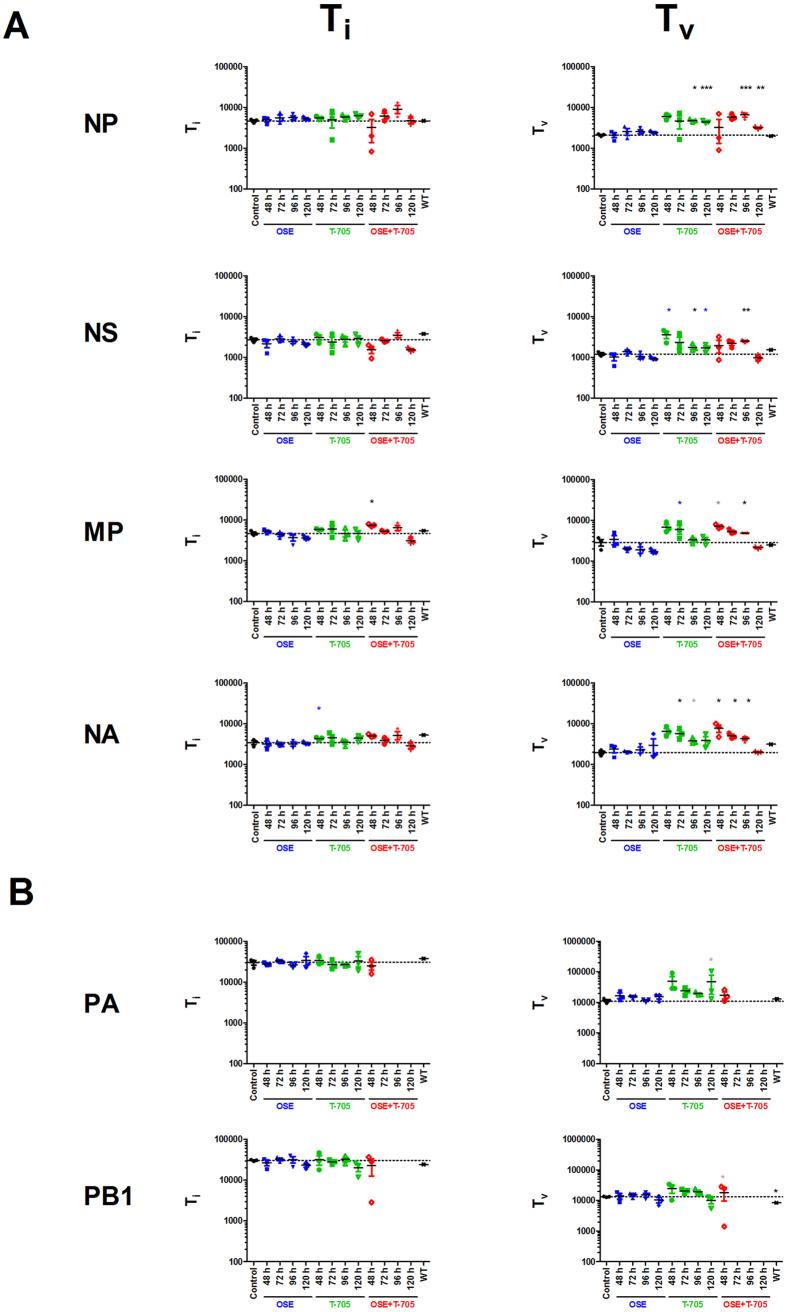
Mutational profile of the virus population in the lungs of A(H5N1) virus–inoculated mice treated with oseltamivir (20 mg/kg/day), T-705 (50 mg/kg/day), or their combination. BALB/c mice were treated as described in the legend for Fig. 1. The charts show the mutational events in the virus population collected from the lung homogenates (*n* = 3/group) at 8 dpi. Transition (T_i_) and transversion (T_v_) events were extracted from the read mappings for the NP, NS, MP, and NA genes (**A**) and the PA and PB1 genes (**B**) and graphed according to treatment group. Data from the groups that received combination therapies initiated at 48, 72, or 120 hpi were excluded from the analyses with PA and PB1 because of the poor sequence coverage and low viral titers obtained. *P < 0.05 and **P < 0.01, compared to control group by 1-way ANOVA. Black * represents statistical significance when compared to control and oseltamivir, blue * represents statistical significance when compared to oseltamivir, and gray * is significant only against control animals. Abbreviations: OSE, oseltamivir; OSE + T-705, oseltamivir and T-705 combination; NP, nucleoprotein; NS, nonstructural; MP, matrix; NA, neuraminidase; PA, acid polymerase; PB1, polymerase basic 1; WT, wild-type virus used for inoculation of mice.

We and other researchers have previously shown that T-705 acts as a purine analogue, resulting in more transition (T_i_) and transversion (T_v_) events, primarily involving the guanosine nucleoside^32^,^39^,^40^. With this in mind, we extracted the T_i_ (A↔G and C↔T substitutions) and T_v_ (A↔C/T and G↔C/T substitutions) events from the deep-sequencing read mappings for the 4 genes (NP, NA, MP, and NS). Except for a few instances in the MP and NS genes, there were essentially no differences in the number of T_i_ events in the treatment groups (Fig. 8A). Strikingly, T-705, whether administered singly or in combination with oseltamivir, increased the T_v_ incidence, compared to that in the oseltamivir-treated and control groups. The T_i_ and T_v_ levels in the oseltamivir-treated group were comparable to the baseline level. To confirm our observation and to validate that it was not restricted to these 4 segments, we reanalyzed our PA and PB1 sequence data from the gene-specific amplification run (Fig. 8B). For comparable analysis, we excluded outlier samples from the combination treatment group that had poor gene coverage and read depths. Consistent with our earlier results, there were no differences in the T_i_ events, whereas the number of T_v_ events was increased in mice that received T-705. These data demonstrate that the virus population showed an increased number of T_v_ events in 6 of the 8 gene segments of the influenza virus genome. Taken together, these results demonstrate that both drugs have independent effects on the viral population, even when administered in combination.

## Discussion

This study is the first to investigate the efficacy of combination therapy with oseltamivir and T-705 against HPAI A(H5N1) virus infection, and, more importantly, the potential of combination therapy to extend the treatment window when treatment is delayed by up to 120 hpi. Although various antiviral agents have been used in combination with NAIs (e.g., amantadine, rimantadine, ribavirin, human IFN-α, plant extracts, and others) for different subtypes of influenza viruses^42^, including the HPAI A(H5N1) viruses^17^,^43^, the major drawback of these studies has been the use of non–FDA-approved drugs (ribavirin, human IFN-α, plant extracts) and the high frequency of resistance to other compounds (amantadine, rimantadine) among circulating human viruses^17^,^43^. The development of T-705 presented an opportunity to use it in a combination therapy regimen. In murine lethal-infection models, T-705 showed strong efficacy against a variety of influenza viruses, including A(H5N1) viruses^21^,^23^,^44^,^45^,^46^, with treatment being delayed by up to 72 hpi^21^, and the efficacy of combined oseltamivir and T-705 against several strains of seasonal influenza viruses as well as mouse-adapted low pathogenic A(H5N1) influenza virus was also investigated in mice^23^,^47^. Extending the treatment window is important if patients admitted to hospital more than 48 h after the onset of symptoms are to be successfully treated, and administering suboptimal doses of drug combinations, as opposed to the optimal dose of a single drug, may not only inhibit virus replication but also reduce the possibility of drug side effects, e.g., by decreasing the mitochondrial toxicity associated with nucleoside analogs^48^,^49^.

We consider that the highly protective efficacy of an oseltamivir and T-705 combination against lethal A(H5N1) infection is achieved because the two drugs affect different stages of the virus replication cycle, thereby preventing the rapid replication and spread of the virus and, in turn, resulting in a reduced virus load and the consequent alleviation of lung pathology and hypercytokinemia. In our study, the lung virus titers of A/Turkey/15/2006 (H5N1) influenza virus in mice peaked on 6 dpi (data not shown), and the innate immune response was also highly responsive around 3 to 5 dpi^50^. We believe that initiating the combination therapy before or around the peak of H5N1 virus replication in the mouse lungs and during a time of active innate immunity was crucial to achieving the most beneficial effect. Cytokine overexpression is a major determinant of the pathogenicity of HPAI A(H5N1) influenza viruses in humans and experimental animal models^3^,^51^,^52^. In our study, A(H5N1) virus-infected, mock-treated mice had the highest levels of MCP-1 and IP-10 chemokines in their lung homogenates. MCP-1 and IP-10 are chemoattractants of macrophages and monocytes that may augment inflammatory responses. Importantly, these results are concordant with the results of previous studies on A(H5N1) infection in humans showing that MCP-1 and IP-10 plasma concentrations were higher in patients with fatal A(H5N1) disease than in patients who survived the infection and that those plasma concentrations correlated with higher viral loads in the respiratory tract^3^,^51^.

A(H5N1) virus causes severe pathology characterized by diffuse alveolar damage, with short disease duration of less than 10 to 12 days in human lungs^53^. Alveolar edema and inflammation were observed in mouse lungs infected with A(H5N1) virus in our study and in previous studies^54^. We used histomorphometry as a novel tool to assess the spread of A(H5N1) virus infection and found that combination therapy reduced the extent of pulmonary lesions, thereby hampering the progression of disease. The lack of active virus infection in this region would also be expected to reduce the release of inflammatory cytokines in the affected areas of the lungs. One limitation of the present study was the use of a single HPAI A(H5N1) strain; it would be interesting to examine the efficacy of combined oseltamivir and T-705 in extending the treatment window for infection by A(H5N1) viruses that differ in their pathogenicity profiles, specifically their growth dynamics in the infected host.

The appearance of drug-resistant viruses is a major concern with any antiviral treatment, as it can greatly affect the outcome. NGS is a novel approach that is increasingly used to understand the evolution of influenza viruses and, given its high sensitivity, has great potential for detecting emerging populations of drug-resistant variants. Here, we used NGS to evaluate the emergence of oseltamivir- and T-705–resistant variants under different treatment conditions. We detected no dominant variant(s) with the hallmark oseltamivir-resistance markers in NA glycoprotein. A variant with a V116A NA substitution that is reportedly associated with reduced susceptibility to oseltamivir^31^ was detected at a low frequency in a single mouse that received oseltamivir therapy and eventually cleared the virus. Overall, we conclude that the risk of oseltamivir-resistant variants emerging under the combination therapy regimen is low.

The antiviral mechanisms of the drugs targeting the polymerase complex are less well characterized than those of NAIs. Limited passage of A/Puerto Rico/8/1934 (H1N1) virus in MDCK cells in the presence of T-705 identified variant viruses with substitutions in the PB1, PA, and PB2-binding regions, though these showed, at most, only a 2-fold increase in IC_50_^55^. Prolonged passage of A(H1N1)pdm09 influenza viruses in the presence of T-705 in MDCK cells failed to isolate any T-705–resistant viruses^32^, suggesting that the polymerase complexes are not susceptible to substitution emergence under the pressure of this drug.

We also extended our NGS studies to the effects of T-705 on the virus population and found increased variability in the virus genome, specifically in segments 5 to 8, in T-705–treated mice but not in oseltamivir-treated animals. This is consistent with the proposed antiviral mechanism for each drug. We previously showed that T-705 treatment was associated with increased base-pairing errors for A-G, C-T, and G-T, all of which are possible if T-705RTP, which acts primarily as a GTP competitor, is incorporated into the plus and minus strands of the viral RNA during replication^32^,^40^. Our results also provide some insights into the mechanism of T-705 action and suggest that T-705 is incorporated into the genome^56^, rather than causing early chain termination^40^, and can induce mutagenesis of the replicating viral genome on a viral population scale.

Ideally, drugs for combination therapy should target different steps in the influenza virus replication cycle, thereby promoting rapid extinction of the virus in the host without affecting the adaptive immunity. Combination therapy comes with caveats, however, such as the possibility of multidrug-resistant influenza viruses emerging, a higher incidence of side effects, and lower efficacies of the drugs used as a result of competitive antagonism. Other undesirable consequences of combination therapy include higher treatment costs and lower adherence to drug regimens than with monotherapy^57^. Thus, combination therapy for influenza not only opens new doors but also poses many challenges. A clinical trial with patients infected with A(H5N1) virus is not possible, but extensive preclinical studies with proof-of-concept and controlled clinical trials in patients with severe seasonal influenza may shed light on other aspects, such as the safety and optimal sequence of antiviral administration. We further advocate the use of deep-sequencing technology for monitoring and early detection of potential drug-resistance mutants.

In conclusion, our findings suggest that combination therapy with virus-targeted antivirals that differ in their mechanism of action, such as oseltamivir and T-705, would be a beneficial strategy for extending the window of treatment opportunity in patients with severe influenza and should be considered an appropriate intervention for clinical testing.

## Methods

### Influenza A(H5N1) virus, cells, and compounds

Highly pathogenic influenza A/Turkey/15/2006 (H5N1) virus (HA clade 2.2.1) was propagated in 10-day-old embryonated chicken eggs for 48 h at 35 °C. Influenza A/Turkey/15/2006 (H5N1) virus was well-characterized previously^43^ and represents clade 2, the HA clade of A(H5N1) viruses that infects humans and continues to evolve rapidly. Madin-Darby canine kidney (MDCK) cells (ATCC, Manassas, VA) were maintained in Eagle’s minimum essential medium (MEM) (Gibco) supplemented with 5% fetal calf serum. The prodrug oseltamivir phosphate (oseltamivir) was dissolved in sterile distilled water, and T-705 was resuspended in ORA-PLUS suspending vehicle (Paddock laboratories, LLC). Oseltamivir and T-705 were provided by F. Hoffmann-La Roche Ltd. (Basel, Switzerland).

### Biosafety, biosecurity, and animal care

All experiments with HPAI A(H5N1) viruses were conducted in an animal biosafety level (ABSL) 3+ containment facility in accordance with USDA 9 CFR 121; 7 CFR 331 and were approved by the Governing Board of the US National Research Council. All experimental protocols involving animals were approved by the Institutional Animal Care and Use Committee (IACUC) of St. Jude Children’s Research Hospital.

### Infectivity of A(H5N1) viruses

The 50% tissue culture infectious dose (TCID_50_) of the A(H5N1) virus was determined in MDCK cells. After 72 h of incubation at 37 °C, the hemagglutination (HA) activity was assayed with 0.5% chicken red blood cells (Rockland Immunochemicals). Titers were calculated^58^ and expressed as mean log_10_TCID_50_/mL ± standard deviation (SD). The dose of virus that was lethal to 50% of mice (MLD_50_) was determined by inoculating groups of five 6-week-old BALB/c mice (weight, 18–20 g) with serial 10-fold dilutions of the virus.

### Assessment of drug efficacy in mice

Female 6-week-old BALB/c mice (The Jackson Laboratories, Bar Harbor, ME) were anesthetized with isoflurane and inoculated intranasally with 10 MLD_50_ (20 TCID_50_/mouse) of influenza A/Turkey/15/2006 (H5N1) virus in 50 μL of PBS. In a pilot study, treatments of mice with a range of oseltamivir (1, 10, 20, and 40 mg/kg/day) and T-705 (50, 100 and 300 mg/kg/day) doses were initiated at 48, 72, 96 and 120 hpi. The dose for each drug eliciting survival rate between 30% and 50% was considered as sub-optimal. Oseltamivir (20 mg/kg/day) and T-705 (50 mg/kg/day) protected 40% and 50% of the mice, respectively, against lethal challenge with A/Turkey/15/2006 (H5N1) influenza virus when initiated at 96 hpi. Hence, these drug doses were selected as sub-optimal doses to study their efficacy in combination treatment (data not shown). To assess drug efficacy, BALB/c mice were given oseltamivir (20 mg/kg/day), T-705 (50 mg/kg/day), or their combination by oral gavage twice daily (12 h apart) for 5 days. Drug treatment was initiated at 48, 72, 96, or 120 hpi. Virus-inoculated mock-treated (control group) mice received sterile distilled water and ORA-PLUS (1:1) on the same schedule. The mice were monitored daily for clinical signs, weight loss, and survival (*n* = 10/group). Animals that showed signs of severe disease (hunched posture and inability to reach out for food and water) and lost 25% or more of their initial weight were euthanized. Mice were weighed at the indicated dpi, and the weight loss or gain for each mouse was calculated as a percentage of its weight before inoculation (at 0 dpi). Additional groups of mice (*n* = 11/group) were inoculated with A/Turkey/15/2006 (H5N1) influenza virus and were treated with drugs as described above. Three mice from each group were sacrificed at 6, 8, and 10 dpi, and the lungs were harvested, rinsed with sterile PBS, homogenized with a TissueLyser system (Qiagen), and resuspended in 1 mL PBS. The suspensions were cleared by centrifugation at 3000 × *g* for 15 min and used for TCID_50_ assays in MDCK cells (with incubation at 37 °C for 3 days). The limit of virus detection was 1.0 log_10_TCID_50_/mL. The endpoint for mortality was extended to 30% initial weight loss for determining virus titers in mouse lungs (approved by IACUC).

### Lung histopathology and immunohistochemistry

At 8 dpi, additional 2 mice in each experimental group were infused via the trachea with 10% neutral buffered formalin (NBF; Thermo Scientific). Their lungs were then collected and fixed by immersion in 10% NBF for at least 7 days before being embedded, sectioned, and stained with hematoxylin and eosin (HE) or subjected to immunohistochemical (IHC) staining with an antibody to influenza A virus nucleoprotein (NP) (US Biological). The extent of pulmonary involvement was quantified by using an Aperio ScanScope XT Slide Scanner to capture digital images of whole-lung sections stained for viral antigen, and the resulting whole-slide images were analyzed using Aperio ImageScope software (Aperio Technologies). Briefly, the total lung field was manually outlined, and areas with lesions were subdivided into 2 zones; those with inactive/resolved infection (containing histologic lesions but negligible viral antigen) and those with active infections (defined as those containing antigen-positive type II pneumocytes, macrophages, and bronchiolar epithelium). The image analysis software provided quantitative measurements of each area. Comparisons of the extent of pulmonary involvement in each treatment group were based on the calculated percentage of the total lung field with lesions (both active and inactive) and on the percentage of the total lesion lung field with active infection.

### Lung cytokine and chemokine analysis

At 6, 8, and 10 dpi, the concentrations of each of 25 cytokines and chemokines were measured in the lung homogenates (*n* = 3/group) by using a MYCTOMAG-70K-PMX MILLIPLEX^®^ MAP mouse cytokine/chemokine panel (Millipore) according to the manufacturer’s instructions. For each cytokine, the standard curve ranged from 3.2 to 10,000 pg/mL. The multiplex plates were read on a Luminex 100/200 analyzer using the xPonent data acquisition and analysis software.

### RNA extractions and deep amplicon sequencing

At 8 dpi, RNA was extracted from lung homogenates with an RNeasy Mini Kit (Qiagen). RNA was amplified by 2-step PCR with the SuperScript III First-Strand Synthesis System (Thermo Scientific) followed by amplification with Phusion DNA polymerase (Thermo Scientific). Gene-specific primers were used to amplify the PB1, PA and NA genes. The primers sequences are available upon request. PCR amplicons were purified on gels or columns by using a QIAquick 96 PCR Purification Kit (Qiagen). Purified amplicons were prepared for sequencing with the Nextera XT DNA Library Prep Kit (Illumina) and sequenced on a MiSeq system (Illumina), using paired-end (2 × 150 bp) sequencing technology. To identify NA-associated resistance markers, analysis was performed with CLC Genomics Workbench 6.5.1 (CLCbio). High-quality reads (Phred score >30) were mapped to the consensus sequences of the A/Turkey/15/2006 (H5N1) virus stock. Variant analysis was performed using the quality-based variant detection algorithm. Briefly, default quality scores were used, and the variant significance thresholds were set to 5% variant frequency, supported by a minimum of 10 reads in both the forward and reverse directions. For the whole-genome mutational profile, a multiplex 8-segment amplification was performed according to the method of Zhou and colleagues^33^. Reads were aligned to their corresponding consensus sequencing by using MOSAIK^59^. The quality of the alignment and visualization of the coverage profiles was assessed with Qualimap^60^. Variant calling was assessed using V-Phaser 2 and V-Profiler^61^. Per-gene nucleotide S_NT_ values were obtained by using the formula 
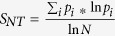
, where *p*_*i*_ is the frequency of a sequence variant and *N* is the length of the corresponding gene. Transition and transversion events were computed directly from the alignment files by using the diversiTools program^62^.

### Statistical analysis

GraphPad Prism 5 software (GraphPad Software, Inc.) was used for statistical analysis. The virus titers and levels of IP-10 and MCP-1 in mouse lungs were compared between treatment and control groups by 2-way analysis of variance (ANOVA). The probability of cumulative survival was estimated by the Kaplan-Meier method and compared between treatment and control groups, and between monotherapy and combination groups by using the log-rank test. One-way ANOVA followed by Bonferroni’s multiple-comparison test was used for analysis of S_NT_ between the treatment and control groups, and between monotherapy and combination groups. A probability value of 0.05 was prospectively chosen to indicate that the findings were not the result of chance alone.

## Additional Information

**How to cite this article**: Marathe, B. M. *et al*. Combinations of Oseltamivir and T-705 Extend the Treatment Window for Highly Pathogenic Influenza A(H5N1) Virus Infection in Mice. *Sci. Rep.* **6**, 26742; doi: 10.1038/srep26742 (2016).

## Supplementary Material

Supplementary Information
