# Supplementary material for: Combinations of Oseltamivir and T-705 Extend the Treatment Window for Highly Pathogenic Influenza A(H5N1) Virus Infection in Mice
Source: Sci Rep. 2016 May 25;6:26742. doi: 10.1038/srep26742 (PMC4879667; doi:10.1038/srep26742)
Supplement: Supplementary Information [file srep26742-s1.pdf]

# Combinations of Oseltamivir and T-705 Extend the Treatment Window for Highly Pathogenic Influenza A(H5N1) Virus Infection in Mice

**Authors:** Bindumadhav M. Marathe[1], Sook-San Wong[1], Peter Vogel[1], Fernando Garcia-Alcalde[2], Robert G. Webster[1], Richard J. Webby[1], Isabel Najera[2], and Elena A. Govorkova[1*]

**Affiliations:**

[1]Department of Infectious Diseases, St. Jude Children's Research Hospital, Memphis, Tennessee.

[2]Roche Pharma Research and Early Development, Infectious Diseases, Roche Innovation Center Basel, F. Hoffmann-La Roche Ltd., Basel, Switzerland.

*To whom correspondence should be addressed: Elena A. Govorkova, Department of Infectious Diseases, St. Jude Children's Research Hospital, 262 Danny Thomas Place, Memphis, TN 38105-3678. Phone: (901) 595-2243. Fax: (901) 595-8559. Email: elena.govorkova@stjude.org

**One-Sentence Summary:** Combination therapy with oseltamivir and T-705 extends the treatment window for H5N1 influenza

# Supplementary data cover page

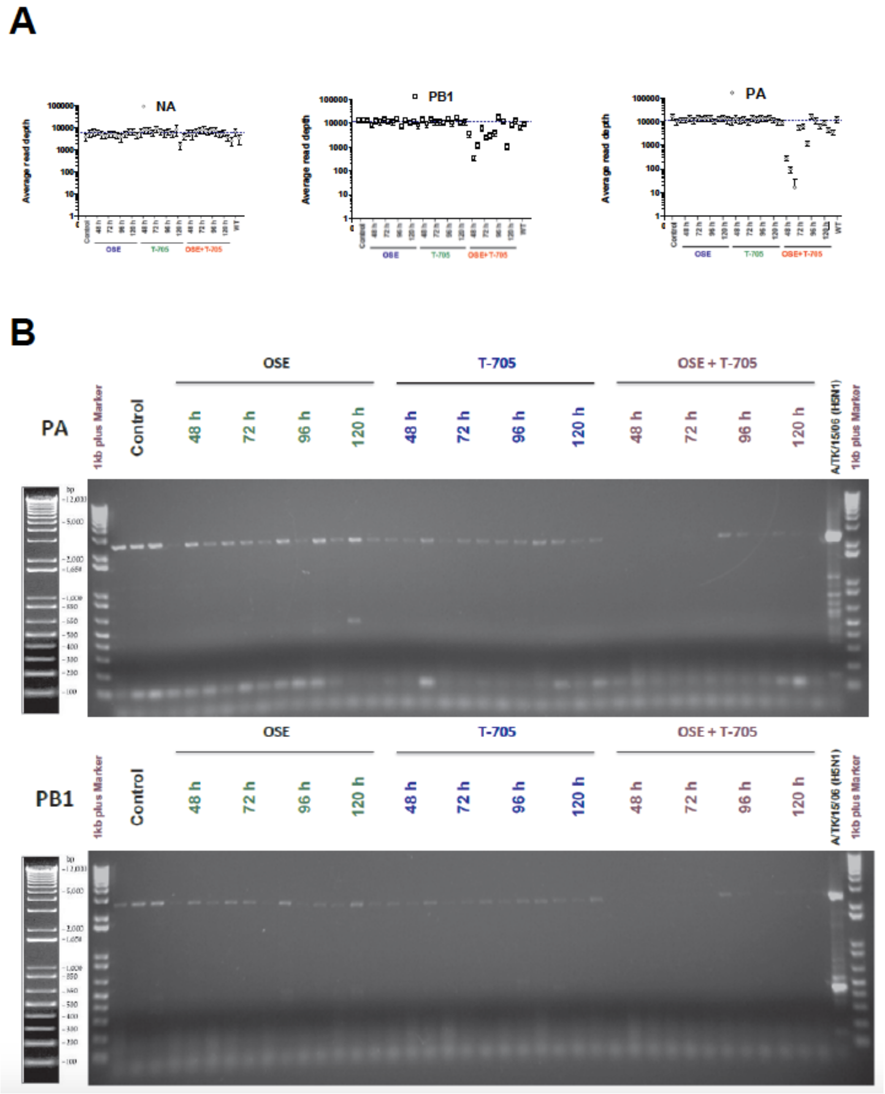


**Supplementary Figure 1. Single-gene PCR amplification of PA and PB1 genes from the lungs of mice inoculated with H5N1 virus and treated with oseltamivir, T-705, or their combination.** BALB/c mice were treated as described in the legend for Fig. 1. RNA was extracted from lungs collected from individual mice ($n$ = 3/group) at 8 dpi. Single-gene amplification was performed for the NA, PB1, and PA genes. The graph shows the mean read depth ± SD for each lung sample for the NA, PB1 and PA genes (A). Full-length amplification of the PA and PB1 genes was performed with gene-specific primers, and PCR products were visualized on a 1% agarose gel (B). The PA and PB1 genes were successfully amplified in viral RNA samples obtained from animals treated with oseltamivir and T-705 at all the assessed time points (48, 72, 96, and 120 hpi). The PA and PB1 genes could not be amplified in the samples obtained from animals treated with the drug combination when treatment was initiated 48 or 72 hpi. Abbreviations: OSE, oseltamivir; OSE + T-705, oseltamivir and T-705 combination; PA, acid polymerase; PB1, polymerase basic 1.

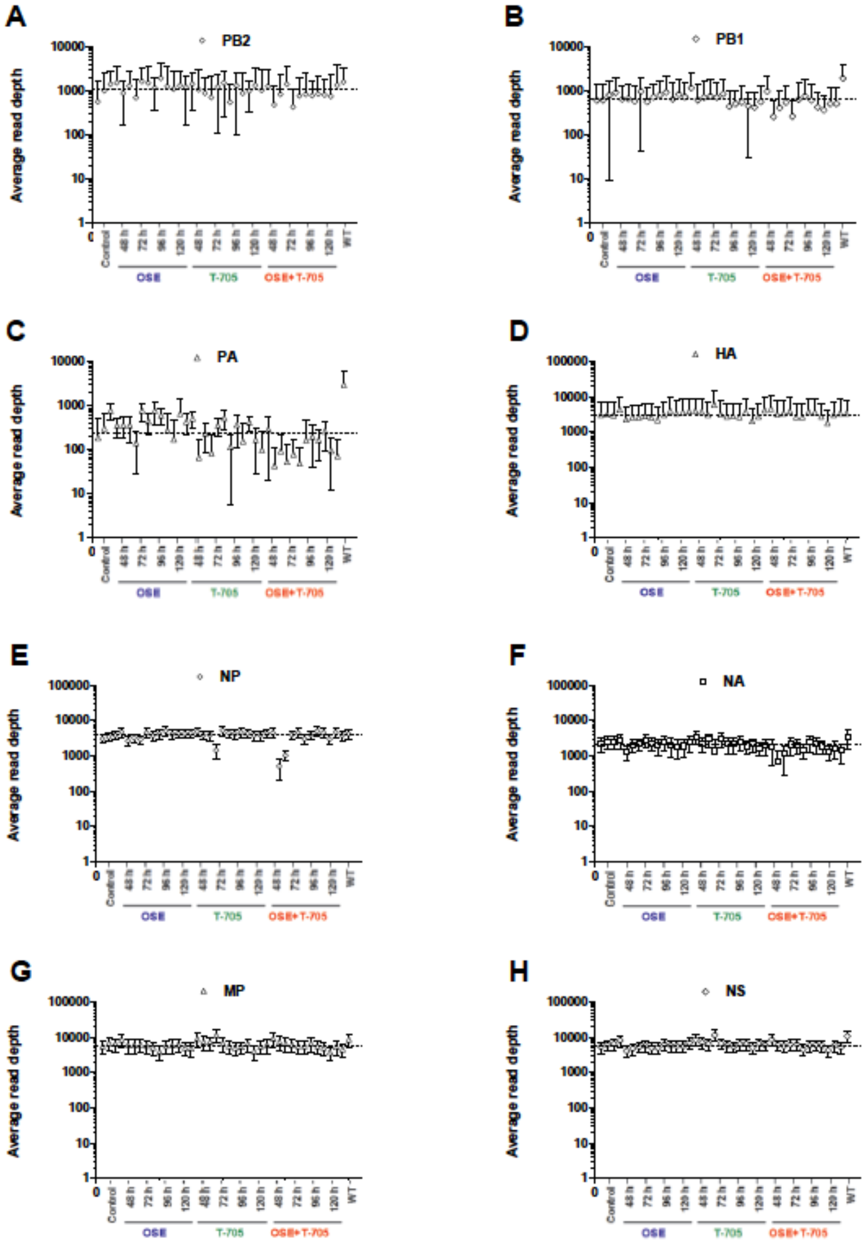


**Supplementary Figure 2. Deep-sequencing coverage across each gene segment during multiplex PCR and single-gene amplification of NA, PA, and PB1.** BALB/c mice were treated as described in the legend for Fig. 1. RNA was extracted from lung homogenates collected from individual mice (*n* = 3/group) at 8 dpi. The mean coverage for PB2 (A), PB1 (B), PA (C), HA (D), NP (E), NA (F), MP (G), and NS (H) obtained from individual animals is presented for each sequencing run. The graphs show the mean read depth ± SD for each lung homogenate sample, and the dashed line indicates the median coverage achieved for all the samples for each gene. Abbreviations: PB2, polymerase basic 2; PB1, polymerase basic 1; PA, acid polymerase; HA, hemagglutinin; NP, nucleoprotein; NA, neuraminidase; MP, matrix; NS, nonstructural.

**Supplementary Table 1. Amino acid changes in the PB1 and PA proteins in the virus population isolated from the lungs of mice inoculated with H5N1 virus and treated with T-705 or oseltamivir–T-705 combination.**

| Mouse ID no. | Treatment group (initiation of treatment) | PB1 | | PA | |
|---|---|---|---|---|---|
| | | Amino acid change | Frequency (variant reads/ total reads) | Amino acid change | Frequency (variant reads/ total reads) |
| 16 | T-705 (48 hpi) | --[a] | -- | T97I<br>S405N | 27.63<br>8.38 |
| 17 | | -- | -- | -- | -- |
| 18 | | -- | -- | -- | -- |
| 19 | T-705 (72 hpi) | M179L<br>G234D | 5.51<br>8.29 | -- | -- |
| 20 | | G37R<br>S98F<br>R151K<br>E159G<br>D617N | 7.21<br>6.99<br>6.08<br>7.87<br>7.32 | -- | -- |
| 21 | | -- | -- | -- | -- |
| 22 | T-705 (96 hpi) | -- | -- | -- | -- |
| 23 | | L10S | 11.11 | -- | -- |
| 24 | | -- | -- | -- | -- |
| 25 | T-705 (120 hpi ) | -- | -- | -- | -- |
| 26 | | -- | -- | -- | -- |
| 27 | | -- | -- | -- | -- |
| 28 | OSE + T-705 (48 hpi) | V170M<br>G304R<br>A659V | 17.8<br>11.92<br>55.86 | E349K | 16.32 |
| 29 | | G540E<br>M174T | 99.57<br>11.6 | L470F<br>A20T<br>P28L<br>T129I | 28.25<br>31.33<br>19.19<br>14.63 |
| 30 | | -- | -- | K643E | 28.57 |
| 31 | OSE + T-705 (72 hpi) | -- | -- | R256K<br>D419N<br>L470F | 7.65<br>6.79<br>7.65 |
| 32 | | P756Q<br>K757N | 5.54<br>6.02 | R212H<br>S247P | 8.96<br>8.83 |
| 33 | | R584C | 96.76 | G316E<br>E410K | 7.59<br>25.41 |
| 34 | OSE + T-705 (96 hpi) | -- | -- | -- | -- |
| 35 | | A144T<br>R260G<br>A547T<br>A547D | 5.19<br>8.8<br>5.05<br>4.04 | -- | -- |

| | | | | | |
|---|---|---|---|---|---|
| | | N671S | 6.3 | | |
| 36 | | L167F | 22.95 | -- | -- |
| 37 | OSE + T-705 (120 hpi) | T291A | 20.76 | -- | -- |
| | | T291V | 1.4 | | |
| | | M348I | 5.37 | | |
| | | M534I | 5.21 | | |
| | | S720P | 9.65 | | |
| 38 | | -- | -- | H41L | 7.18 |
| 39 | | A448T | 42.6 | E206G | 7.44 |
| | | V451I | 5.48 | P271S | 7.3 |
| | | E638K | 10.17 | H326P | 7.75 |

PA, acid polymerase; PB1, polymerase basic 1.
[a] --, no PB1 or PA mutations detected.

PB1 and PA were amplified with gene-specific primers, sequenced, and analyzed as described in the Materials and Methods section. Amino acid substitutions specific to the T-705 treatment groups are shown. The presented mutations were detected with a frequency greater than 5% in the viral population.
